# Supplementary material for: Generation and Characterization of Native and Sialic Acid-Deficient IgE
Source: Int J Mol Sci. 2022 Nov 3;23(21):13455. doi: 10.3390/ijms232113455 (PMC9657026; doi:10.3390/ijms232113455)
Supplement: Supplementary file 1 [file ijms-23-13455-s001.zip › ijms-1894647-supplementary.pdf]

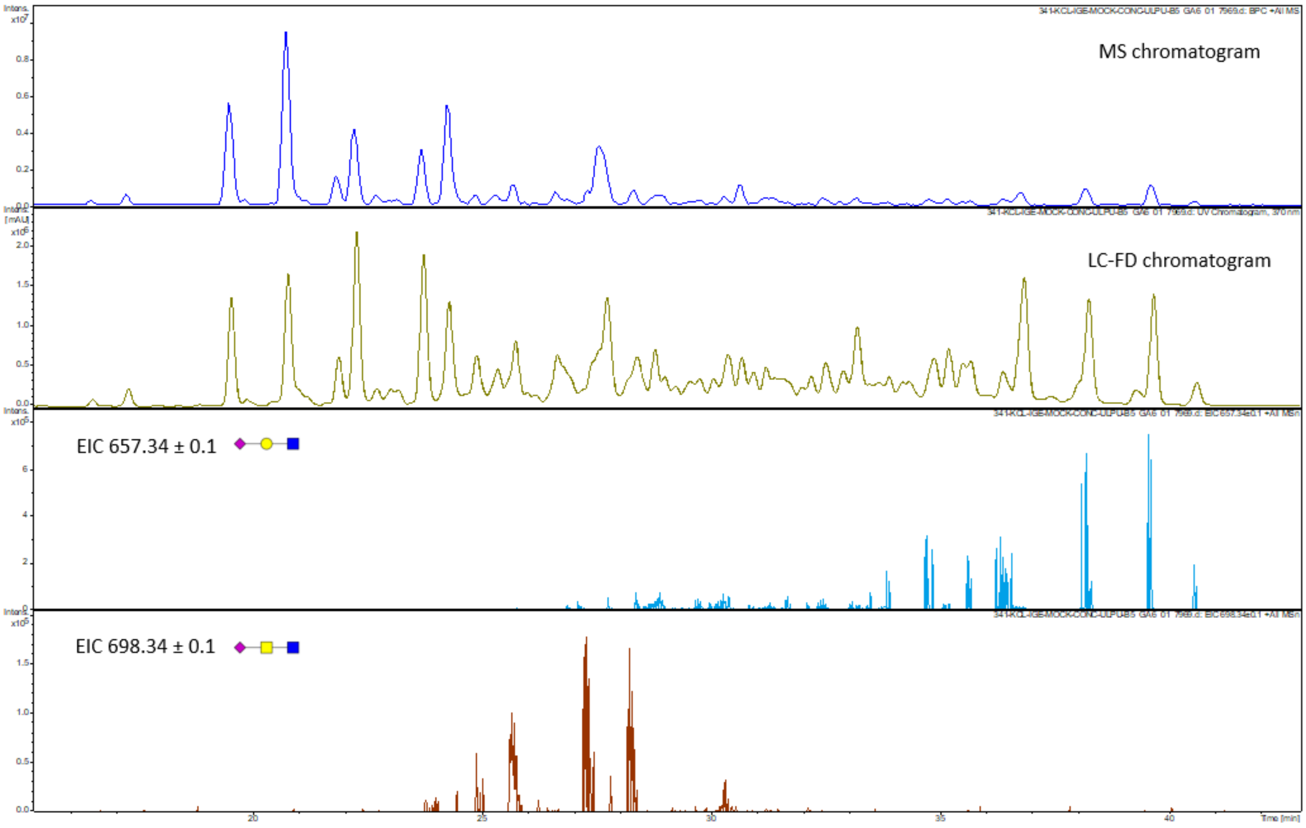


**Supplementary Figure S1.** Extracted Ion Chromatograms for Con-IgE sample [Figure 3; Table 1].

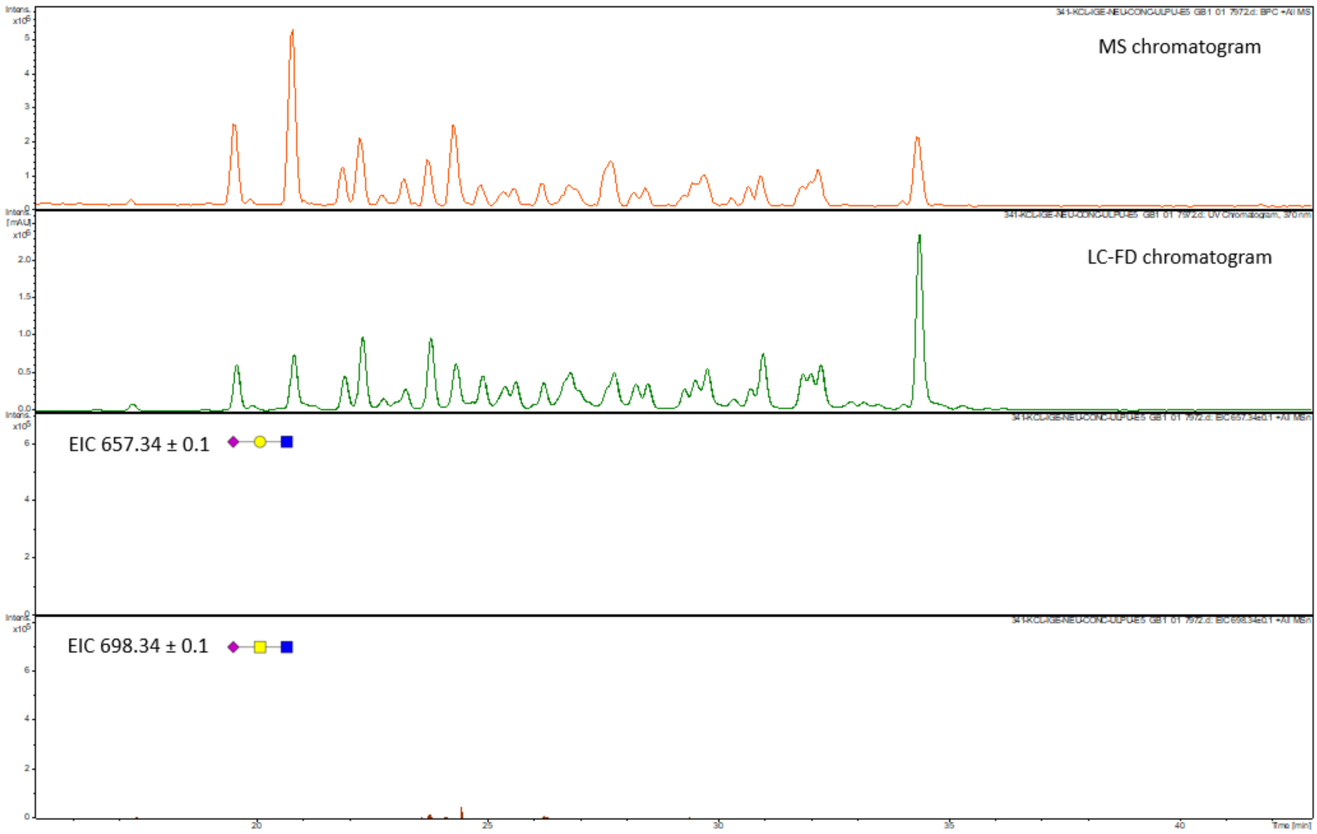


**Supplementary Figure S2.** Extracted Ion Chromatograms for Neu-IgE sample (Figure 3; Table 2).

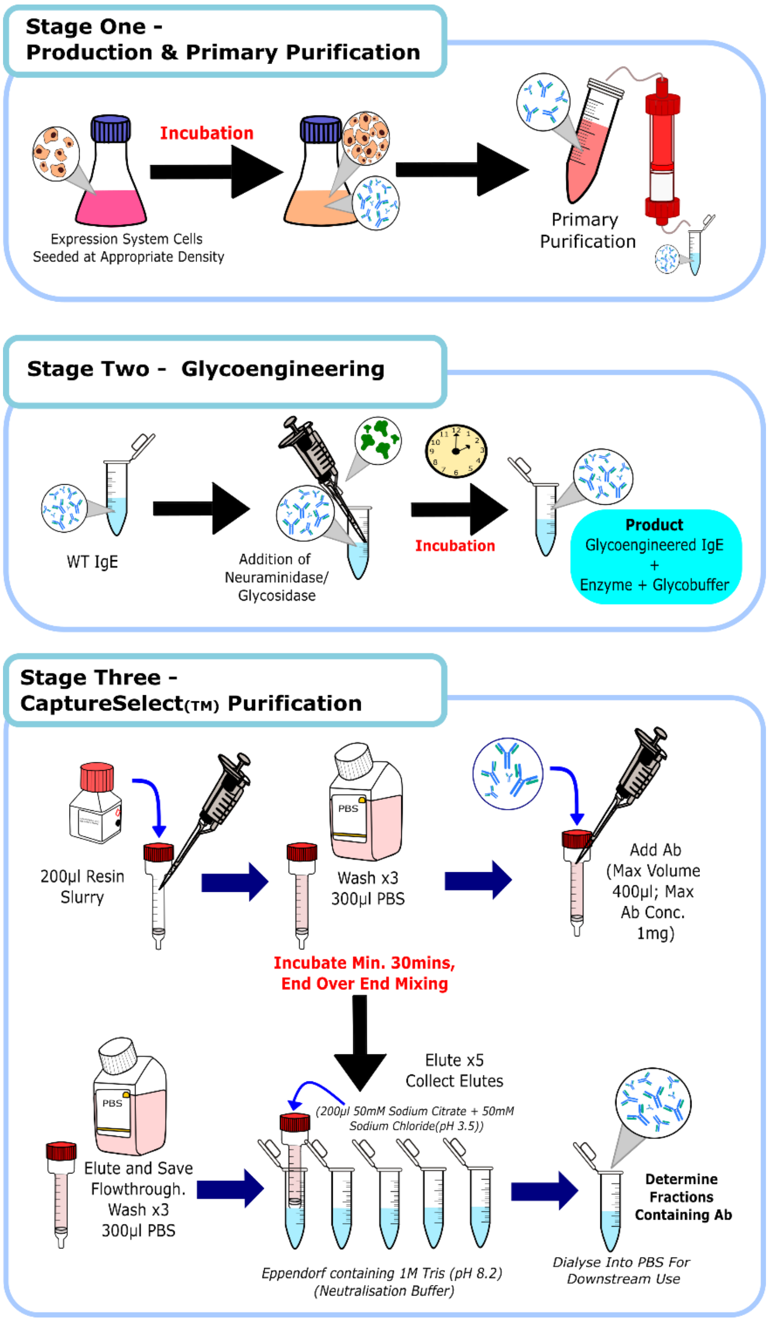


**Supplementary Figure S3.** Complete Pipeline for the Production and Purification of Glyco-engineered IgE Antibody.
